# High efficacy of an in-house antigen B ELISA for diagnosing human cystic echinococcosis compared with a commercial kit and a cell-free DNA assay

**DOI:** 10.1038/s41598-026-58120-x

**Published:** 2026-06-16

**Authors:** Maryam Hataminejad, Reza Valadan, Shahabeddin Sarvi, Seyed Abdollah Hosseini, Alireza Rafiei, Shirzad Gholami

**Affiliations:** 1 Student Research Committee, Mazandaran University of Medical Sciences, Sari, Mazandaran Iran; 2 Toxoplasmosis Research Center, Communicable Diseases Institute, Mazandaran University of Medical Sciences, Sari, Mazandaran Iran; 3 Department of Immunology, Molecular and Cell Biology Research Center, School of Medicine, Mazandaran University of Medical Sciences, Sari, Iran; 4 Department of Parasitology, School of Medicine, Mazandaran University of Medical Science, Sari, Mazandaran Iran

**Keywords:** Cystic echinococcosis, Antigen B, ELISA, Cell-free DNA, Biological techniques, Biotechnology, Diseases, Immunology, Medical research, Microbiology, Molecular biology

## Abstract

Cystic echinococcosis (CE) continues to pose a serious zoonotic health risk in endemic areas. Serodiagnosis, while commonly utilized, faces challenges such as antigenic variability and stage-dependent sensitivity. The purpose of this study was to develop and evaluate an in-house ELISA using the isolated 12-kDa subunit of Antigen B (AgB) from *Echinococcus granulosus* sensu stricto, as well as to compare its performance to that of a commercial ELISA kit and acell-free DNA (cfDNA)-based PCR test. The 12-kDa AgB component was isolated from sheep hydatid cyst fluid and used to create an in-house IgG ELISA. The diagnostic performance was tested using ROC analysis. A total of 51 surgically and CT-confirmed CE patients and 50 healthy controls were included. Plasma cfDNA was collected and amplified using PCR targeting the COX1 gene. Cohen’s kappa and Spearman’s correlation were used to determine agreement. The in-house 12-kDa AgB ELISA demonstrated a sensitivity of 90.2%, a specificity of 100%, and an AUC of 0.936. Similarly, the commercial ELISA kit showed a sensitivity of 92.2% and a specificity of 100%. For its part, cfDNA-PCR successfully identified 88.2% of cases and exhibited a specificity of 100%. There was excellent agreement between the in-house and commercial ELISA kits (κ = 0.878) and serological and molecular techniques (κ = 0.78–0.81). The in-house 12-kDa AgB ELISA is a highly reliable and cost-effective diagnostic tool for CE, with performance equivalent to commercial ELISA kits and cfDNA-PCR assays. Future work should prioritize developing standardized recombinant subunits to improve the reliability and accessibility of AgB-based diagnostics in endemic areas. Moreover, direct cyst-tissue DNA warrants evaluation as a more sensitive and stable template for molecular confirmation, potentially overcoming limitations of serological and plasma cfDNA approaches.

**Supplementary Information:**

The online version contains supplementary material available at https://doi.org/10.1038/s41598-026-58120-x.

## Introduction

Cystic echinococcosis (CE) is a worldwide zoonotic disease caused by *Echinococcus granulosus* sensu stricto, with genotypes G1–G3 being the primary agents of human infection^1,2^. The disease demonstrates a varied geographical distribution, with high prevalence in pastoral communities across South America, East Africa, Central Asia, China, and the Mediterranean Basin^3,4^. The parasite’s life cycle alternates between canids as definitive hosts and domestic animals as intermediate hosts, with humans inadvertently serving as accidental intermediate hosts by consuming parasite eggs. Hydatid cysts are typically found in the liver (70%) and lungs (20%), but can affect any organ, leading to a wide range of clinical symptoms and a challenging diagnosis process^5,6^.

Ultrasonography, computed tomography, and magnetic resonance imaging are the primary imaging modalities used to diagnose CE^7,8^. The WHO-Informal Working Group for Echinococcosis (WHO-IWGE) cyst classification system is based on ultrasonography, which divides cysts into three stages: active (CE1-CE2), transitional (CE3a-CE3b), and inactive (CE4-CE5)^9,10^. However, imaging alone cannot consistently distinguish CE from other benign or malignant cystic lesions, nor can it dependably detect early, small, or extrahepatic cysts^9,11^. Serologic assays continue to be important imaging aids and are frequently employed in clinical and epidemiological settings. Several methods have been employed for CE diagnosis, including IHA, IFA, CCIEP, and ELISA, with ELISA being the most often used due to its practicality, scalability, and favorable sensitivity profile^12,13^. However, the performance of serological assays is critically dependent on the quality and purity of the antigen source. While native hydatid cyst fluid (HCF) exhibits high sensitivity, its complex composition, which includes host-derived proteins and a heterogeneous mixture of parasitic antigens, often compromises specificity and can lead to cross-reactivity with other helminthic infections^14^.

Among HCF components, antigen B (AgB) is the most abundant and diagnostically relevant lipoprotein complex. AgB is a thermostable multimeric protein of ~ 120–160 kDa that dissociates under reducing conditions into characteristic subunits of approximately 8–12, 16, and 24 kDa. It is encoded by a multigene family (EgAgB1–EgAgB5), whose isoforms exhibit notable sequence diversity^15,16^. Previous research has demonstrated that the 12-kDa subunit, in particular, exhibits strong immunoreactivity, high specificity, and reduced cross-reactivity, making it one of the most reliable single antigenic components for ELISA-based CE diagnosis^17^.

During the past decade, advances in molecular diagnostics have provided new tools to complement serology. The detection of circulating cell-free DNA (cfDNA) of *E. granulosus* in plasma, serum, and urine offers a promising minimally invasive biomarker that may identify early infections, cyst leakage, or ruptured cysts^18^. While early studies indicate that cfDNA assays can detect infections independent of host antibody production, their sensitivity remains limited by low circulating DNA levels and technical constraints, particularly in resource-limited settings. Accordingly, their role in routine CE diagnosis requires further evaluation alongside established serologic methods^19^. In the present study, we established an in-house ELISA using purified AgB (12-kDa fraction) and evaluated its diagnostic performance in human CE. The assay’s results were compared with a commercial ELISA kit (Pishtaz Teb, Iran Co.) as well as the cfDNA PCR method.

## Materials and methods

### Sample collection and HCF preparation

HCF was obtained from naturally infected sheep livers at the Sari municipal abattoir under veterinary supervision and according to biosafety regulations. Only intact, uncontaminated cysts were selected. The liver surface was disinfected with ethanol, and cyst fluid was aseptically aspirated. The HCF was clarified by centrifugation at 3000×*g* for 15 min at 4 °C, after which the supernatant was aliquoted and stored at − 80 °C for subsequent antigen preparation^20^.

### Genotyping of hydatid cyst isolates

Protoscoleces were aseptically collected from fertile hydatid cysts of sheep livers and washed three times with PBS (pH 7.4). Genomic DNA was isolated from ~ 20 mg of sedimented protoscoleces using a commercial DNA Isolation Kit (Dena Zist, Iran), and DNA quantity and purity were assessed with a spectrophotometer (Thermo Fisher Scientific, USA).

For molecular genotyping of *E. granulosus* s.s., partial fragments of the mitochondrial COX1 and NAD1 genes were amplified using previously published species-specific primers (COX1: F 5′–TTTTTTGGGCATCCTGAGGTTTAT–3′, R 5′–TAAAGAAAGAACATAATGAAAATG–3′; NAD1: F 5′–AGATTCGTAAGGGGCCTAATA–3′, R 5′–ACCACTAACTAATTCACTTTC–3′). Primer specificity was confirmed via in-silico BLAST analysis^21,22^.

The PCR reactions (50 µL) consisted of master mix (Ampliqon, Denmark), primers (10 pmol/µL each), 4 µL of DNA template, and nuclease-free water (Thermo Fisher Scientific, USA).

Amplification was performed in a thermal cycler (Bio-Rad, USA), with the following protocol: initial denaturation at 94 °C for 5 min; 35 cycles of denaturation at 94 °C for 30 s, annealing at 55 °C for 45 s, and extension at 72 °C for 1 min; followed by a final extension at 72 °C for 10 min. The PCR products were visualized on a 1.5% agarose gel (Sigma-Aldrich, USA) using Safe DNA Gel Stain (Biotium, USA). For *E. granulosus* s.s., the expected amplicon sizes were ~ 450 bp for the cox1 gene and ~ 450 bp for the nad1 gene. A positive control (DNA from a confirmed *E. granulosus* cyst) and a no-template control were included in all PCR runs. Purified amplicons were subjected to unidirectional Sanger sequencing (Gene Fanavaran Co., Tehran, Iran). Sequences were edited in BioEdit 7.2 and compared with GenBank reference sequences using BLAST to confirm the genotype of *E. granulosus* s.s. isolates.

### AgB purification and characterization

AgB was purified from the clarified HCF according to the method of Oriol et al.^23^. Briefly, 100 mL of HCF was dialyzed overnight against 5 mM acetate buffer (pH 5.0) at 4 °C, centrifuged at 50,000×*g* for 30 min, and resuspended in 0.2 M phosphate buffer (pH 8.0). Ammonium sulfate precipitation was used to remove globulins, and heat-stable AgB was isolated by boiling for 15 min and centrifugation at 50,000×*g* for 60 min. The antigen was then extracted from the supernatant. The protein content was measured using the Bradford assay. Purification and molecular profile were verified by SDS-PAGE under reducing conditions, revealing a prominent ∼12-kDa band characteristic of AgB. Sodium Azide (NaN₃, 0.02% w/v) was added to the final antigen, which was then stored in sterile vials at − 80 °C for subsequent assays^23^.

### Study design and patient samples

This study utilized 51 blood samples from surgically and CT-confirmed CE patients, collected between March 2023 and August 2025 at the Mazandaran Registry Center for Hydatid Cyst (MRCHC). Following processing, serum was allocated for ELISA testing, and plasma was reserved for cfDNA extraction and PCR analysis. All samples were stored at − 80 °C until use. Demographic and clinical data were collected, including age, gender, residence, cyst number and organ, cyst type, and cyst stage. For negative controls, 50 blood samples were collected from healthy neonates who had no clinical or serological signs of exposure to *Echinococcus* spp. To assess cross-reactivity, 20 serum samples from patients with other parasitic infections were included, covering *Toxoplasma gondii* (*n* = 4), *Lophomonas blattarum* (*n* = 2), *Leishmania major* (*n* = 2), *Strongyloides stercoralis* (*n* = 3), *Hymenolepis nana* (*n* = 2), *Entamoeba histolytica* (*n* = 2), *Fasciola hepatica* (*n* = 2), and *Ascaris lumbricoides* (*n* = 3).

### In-house 12-kDa AgB ELISA

An in-house ELISA was established utilizing the purified 12-kDa AgB subunit as the capture antigen. Microtiter plates (Thermo Fisher Scientific, USA) were incubated overnight at 4 °C with antigen doses of 2.5, 5, 10, 20, and 40 µg/mL in carbonate-bicarbonate buffer (pH 9.6). A coating concentration of 20 µg/mL was selected for its best signal-to-noise ratio. The plates were then incubated with 5% bovine serum albumin (Sigma-Aldrich, USA) in phosphate-buffered saline with 0.05% Tween-20 (PBST) for 1.5 h at 37 °C. Patient sera, diluted 1:100 in blocking buffer, were administered in duplicate and incubated for one hour at 37 °C. After three washes with PBST, horseradish peroxidase (HRP)-conjugated goat anti-human IgG (Biosciences, USA; diluted 1:10000) was introduced and incubated for one hour at 37 °C. Following an additional washing step, the reaction was developed with a 3,3’,5,5’-tetramethylbenzidine (TMB) substrate solution (Biosciences, USA) for 15 min in the dark. The enzymatic reaction was terminated by the addition of 2 M sulfuric acid, and optical density was measured at 450 nm with a reference filter of 630 nm utilizing an ELISA plate reader (BioTek, USA). The diagnostic cut-off value was determined by receiver operating characteristic (ROC) curve analysis, applying the Youden index. For comparative purposes, all serum samples were also analyzed in parallel using a commercial *E. granulosus* IgG ELISA kit (Pishtaz Teb, Iran) according to the manufacturer’s instructions.

### cfDNA extraction and PCR amplification

cfDNA was extracted from 200 µL of plasma according to the manufacturer’s protocol using the Sambio™ DNA Minikit. DNA concentration and purity were measured with a NanoDrop™, and samples were subsequently stored at − 20 °C. Molecular detection focused on the *E. granulosus* COX1 mitochondrial gene with species-specific primers (COX1-F: 5′–TTTTTTGGGCATCCTGAGGTTTAT–3′; COX1-R: 5′–TAAAGAAAGAACATAATGAAAATG–3′), which were verified in silico through BLAST analysis. The PCR reactions (25 µL) comprised master mix, primers (10 pmol/µL each), 5 µL cfDNA, and nuclease-free water. Thermal cycler: 94 °C for 5 min; 35 cycles of 94 °C for 30 s, 55 °C for 45 s, and 72 °C for 1 min; final extension at 72 °C for 7 min. The products were seen on a 1.5% agarose gel using Safe DNA Gel Stain. A ~ 450 bp band confirmed *E. granulosus* s.s. Positive (DNA from a confirmed hydatid cyst) and negative (no template) controls were included in all runs.

### Quality control and assay validation

To verify the reliability and repeatability of the in-house AgB ELISA, quality control and validation procedures were carried out. Precision was assessed utilizing intra- and inter-assay analysis on high- and low-positive sera. Each control sample was tested in ten duplicates within one plate for intra-assay precision, while inter-assay precision was determined by testing the identical controls in duplicate over ten consecutive runs. The coefficients of variation (CV%) were computed as (SD/mean OD) × 100, with acceptable limits of < 10% for intra-assay and < 15% for inter-assay variability. Assay linearity was subjected to sequential two-fold dilutions using PBS to create a concentration range from 200 to 0.8 µg/mL. Each dilution was tested in the in-house AgB ELISA, and the OD was measured at 450 nm. Observed OD values were plotted against expected values, and a correlation coefficient (R^2^ ≥ 0.98) was considered indicative of strong linearity.

### Statistical analysis

Statistical analyses were conducted using IBM SPSS Statistics version 26.0 (Armonk, NY, USA) and GraphPad Prism version 10.0 (San Diego, CA, USA). Continuous data are presented as mean ± standard deviation (SD) and categorical variables as frequencies. Chi-square analysis was used for categorical comparisons of clinical variables and serological status, as the exploratory binary logistic regression analysis revealed no significant associations. Diagnostic performance of AgB ELISA and the commercial ELISA kit was evaluated by calculating sensitivity, specificity, positive predictive value (PPV), negative predictive value (NPV), overall accuracy, and ROC curve analysis. The optimal cut-off value was determined using the Youden index and maximal accuracy, and AUC was reported. Correlation and agreement between diagnostic methods were assessed using Spearman’s rank correlation for OD values and Cohen’s kappa for binary outcomes. Kappa values were interpreted as follows: 0.00–0.20 (poor), 0.21–0.40 (fair), 0.41–0.60 (moderate), 0.61–0.80 (good), and 0.81–1.00 (excellent). Group differences were analyzed with the independent samples t-test, and statistical significance was defined as *p* < 0.05.

## Results

### Protoscolex isolation and molecular genotyping of hydatid cysts

Viable protoscoleces, isolated from fertile sheep hydatid cysts, were used for molecular genotyping. Amplification of the mitochondrial cox1 (~ 450 bp) and nad1 (~ 400 bp) gene fragments yielded distinct, single bands with no nonspecific amplification (Supplementary 1).

**Fig. 1:**
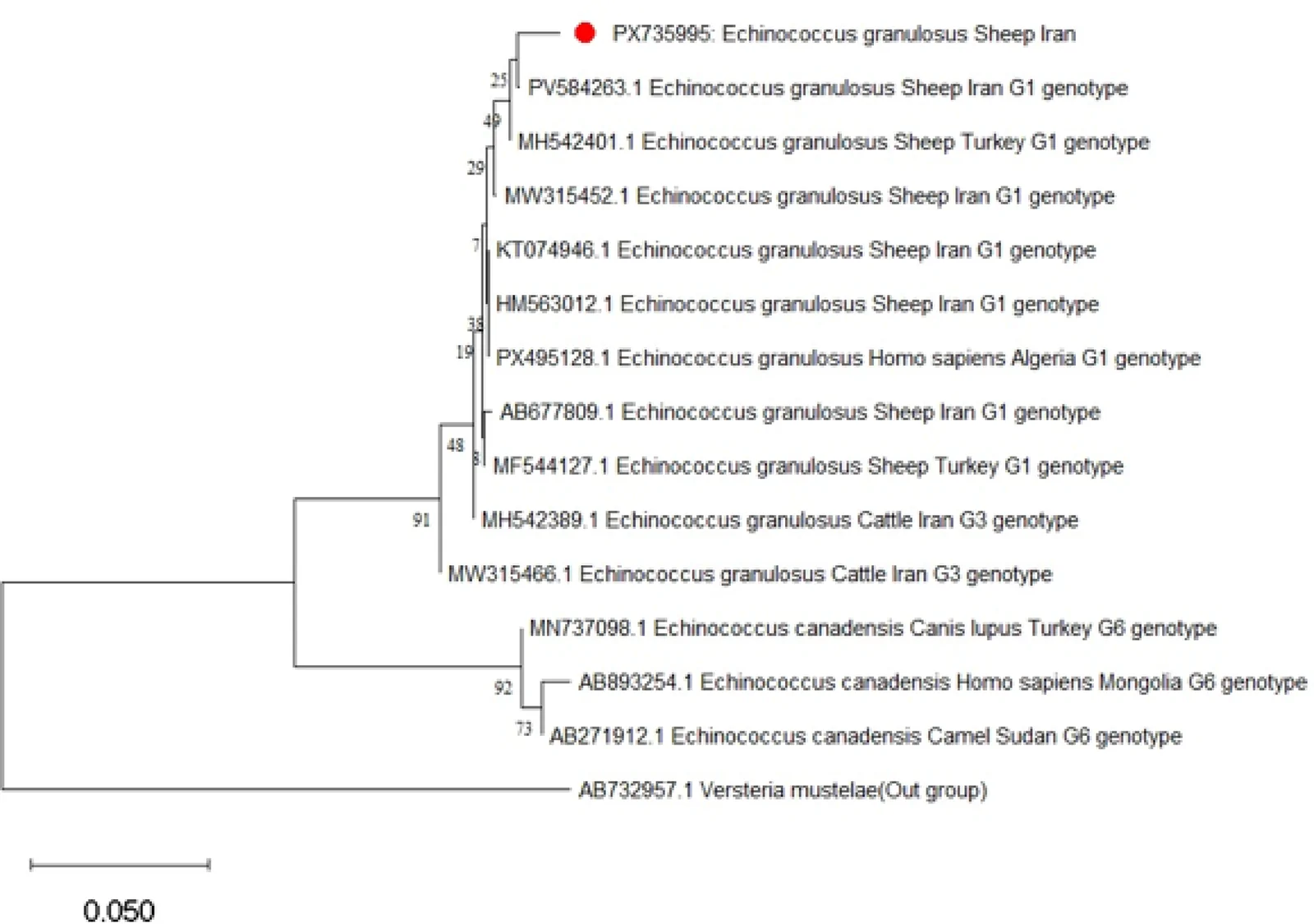
Phylogenetic tree based on mitochondrial cox1 gene sequences (~ 450 bp) of protoscoleces isolated from sheep hydatid cysts. The tree illustrates the clustering of the isolate, represented by a red circle (), within the *E. granulosus* s.s. G1 genotype clade, supported by high bootstrap values. Reference sequences of various Echinococcus genotypes and *Versteria mustelae* (outgroup) are included for comparison.

**Fig. 2:**
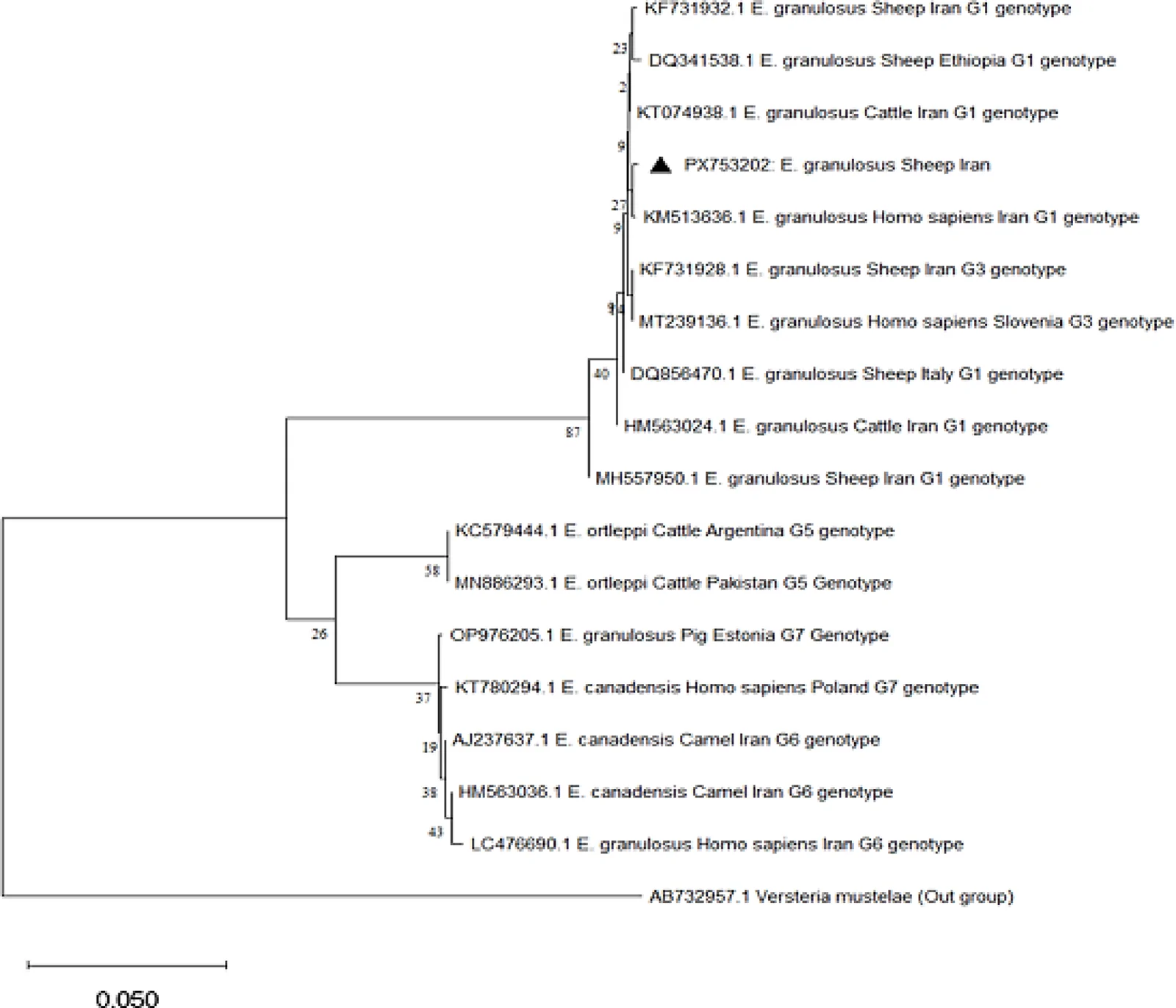
Phylogenetic tree based on mitochondrial nad1 gene sequences (~ 400 bp) of protoscoleces isolated from sheep hydatid cysts. The tree illustrates the clustering of isolate, represented by a black triangle (▲), within the *E. granulosus* s.s. G1 genotype clade, supported by high bootstrap values. Reference sequences of various Echinococcus genotypes and *Versteria mustelae* (outgroup) are included for comparison.

Sequencing and BLAST analysis of representative isolates demonstrated > 99% identity with reference sequences for *E. granulosus* s.s. (genotype G1) (e.g., GenBank accession numbers PX735995 for cox1 and PX753202 for nad1). Phylogenetic clustering confirmed that all isolates belonged to the *E. granulosus* s.s. lineage, with no evidence of mixed or non-G1 genotypes in the sampled region (Figs. 1 and 2).

### SDS-PAGE analysis and characterization of purified AgB

SDS-PAGE analysis of the purified AgB preparation revealed a distinct protein band at approximately 12-kDa, representing the principal subunit of AgB (Supplementary 2). The absence of high-molecular-weight impurities confirmed the successful purification and enrichment of the 12-kDa subunit. Protein quantification by the Bradford assay indicated an average concentration of 940 µg/mL.

### Demographic profile and clinical–serological associations

A total of 51 surgically and CT-confirmed CE patients were included in the analysis, consisting of 24 males (47.1%) and 27 females (52.9%), with a mean age of 46.2 ± 15.5 years (range: 10–76). More than half of the participants were urban residents (54.9%). Hepatic involvement was predominant (68.6%), followed by pulmonary cysts (31.4%). According to the WHO-IWGE classification, cyst stages were distributed as follows: CE1 (21.6%), CE2 (27.5%), CE3 (21.6%), CE4 (17.6%), and CE5 (11.8%). Nearly half of the cysts were classified as active (49.0%), while 21.6% were transitional and 29.4% were inactive. Most patients (70.6%) presented with a single cyst. No significant associations were found between demographic variables and cyst localization (*p* > 0.05, Table 1).

**Table 1:** Demographic and clinical characteristics of patients with CE.

Variable	Category	Number	Percent (%)	*p*-value
Sex	Male	24	47.1	0.61
	Female	27	52.9	
Residence	Urban	28	54.9	0.48
	Rural	23	45.1	
Organ involved	Liver	35	68.6	0.07
	Lung	16	31.4	
Cyst stage (WHO)	CE1	11	21.6	0.85
	CE2	14	27.5	
	CE3	11	21.6	
	CE4	9	17.6	
	CE5	6	11.8	
Cyst activity	Active	25	49.0	0.92
	Transitional	11	21.6	
	Inactive	15	29.4	
Number of cysts per patient	1 Cyst	36	70.6	0.78
	2 Cysts	12	23.5	
	3 Cysts	2	3.9	
	4 Cysts	1	2.0	

The serological performance of the commercial ELISA kit and the AgB-ELISA was assessed across four clinical variables: organ involvement, cyst stage, cyst activity, and cyst number. Both assays demonstrated comparable response patterns. Regarding organ involvement, a small number of hepatic cases (3 in the commercial ELISA kit and 4 in the AgB-ELISA) and one pulmonary case (in both assays) were seronegative. Across cyst stages, both tests showed high positivity rates, particularly in CE3–CE5 cysts. Based on the results, the AgB-ELISA showed lower sensitivity for active cysts (4/25 negative) compared to the commercial kit (3/25 negative). Both kits performed identically for transitional cysts (1/11 negative). Notably, no negative results were observed for inactive cysts with either ELISA method. Among patients with single cysts, the commercial ELISA kit and AgB-ELISA yielded 4/36 and 5/36 negative results, respectively; all patients with multiple cysts were seropositive. Chi-square analyses revealed no significant association between serological outcomes and any clinical variable (*p* > 0.05). Thus, cyst localization, stage, biological activity, and number did not significantly affect reactivity in either the commercial ELISA kit or the AgB-ELISA within this cohort (Table 2).

**Table 2:** Association between clinical variables and serological results in confirmed CE patients (*n* = 51).

Variable	Category	AgB-ELISA positive (*n*)	AgB-ELISA negative (*n*)	CI 95%	*p*-value	Commercial ELISA kit positive (*n*)	Commercial ELISA kit negative (*n*)	CI 95%	*p*-value
Cyst-Organ	Liver	31	4	0.008–2.887	0.210	32	3	0.009–5.111	0.345
	Lung	15	1			15	1		
Cyst-Stage	CE1	9	2	0.14–1.701	0.128	9	2	0.005–1.701	0.109
	CE2	12	2			13	1		
	CE3	10	1			10	1		
	CE4	9	0			9	0		
	CE5	6	0			6	0		
Cyst-Status	Active	21	4	0.259–14.612	0.518	22	3	0.329–45.490	0.282
	Transitional	9	1			10	1		
	Inactive	15	0			15	0		
Cyst-Number	Single	31	5	0.735–1.436	0.998	32	4	0.750–1.471	0.998
	Double	12	0			12	0		
	Triple	2	0			2	0		
	Quadruple	1	0			1	0		

### Diagnostic performance of the in-house 12-kDa AgB ELISA

Optimization experiments demonstrated that a coating concentration of 20 µg/mL provided the highest signal-to-noise ratio with minimal background reactivity. Using this optimized condition, sera from 51 CE patients and 50 healthy controls were analyzed.

**Fig. 3:**
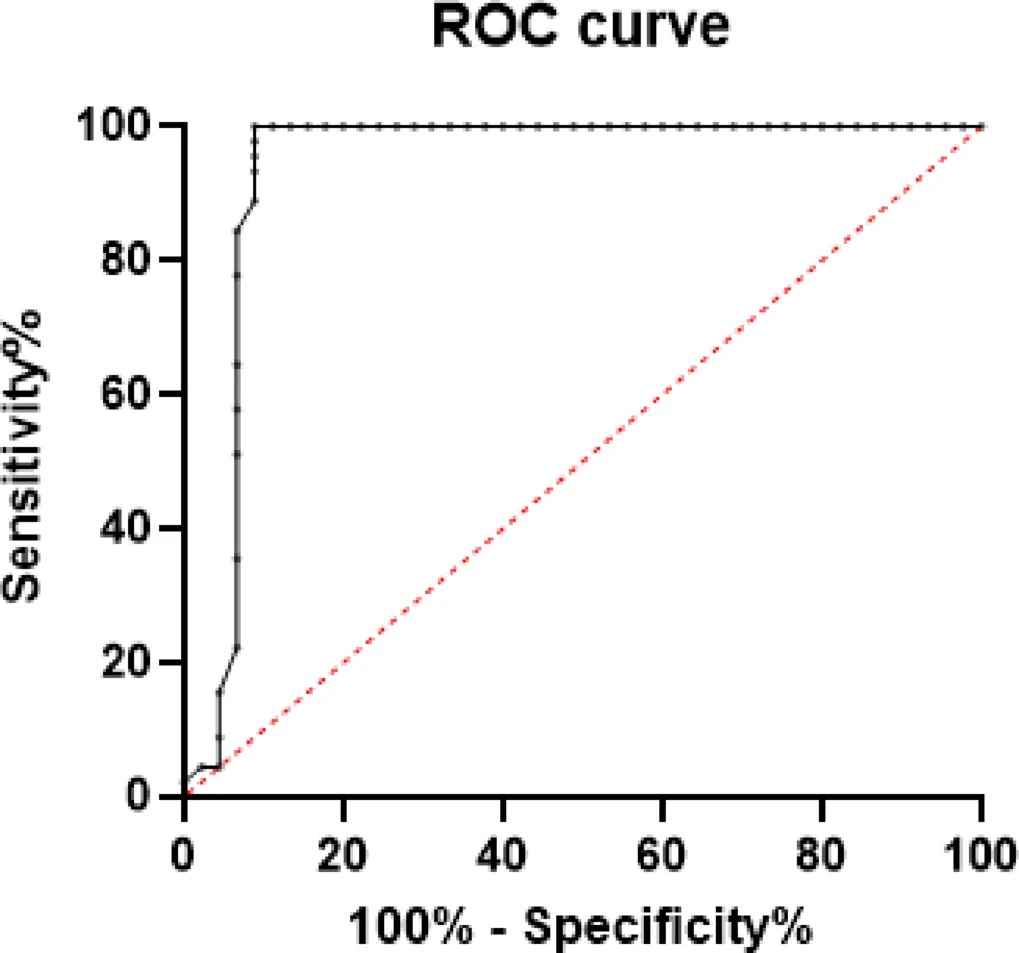
ROC curve for the AgB-ELISA (AUC = 0.936). The dot indicates the optimal cut-off (OD = 0.256), yielding 90.2% sensitivity and 100% specificity.

The mean OD_450_ for CE-positive sera was 0.8489 ± 0.314 (range: 0.265–2.03), whereas the mean OD_450_ for healthy controls was 0.051 ± 0.004 (range: 0.042–0.059). The difference between groups was significant (*p* < 0.001). ROC analysis indicated excellent diagnostic discrimination with AUC = 0.936 ± 0.021 (95% CI: 0.895–0.978, Fig. 3). The optimal cutoff determined by the Youden index was 0.256. Using this cutoff (OD ≥ 0.256 = positive), the assay was achieved. At this threshold, the assay identified 46 true positives and 50 true negatives, with 5 false negatives and no false positives, resulting in a sensitivity of 90.2%, specificity of 100%, positive predictive value of 100%, negative predictive value of 90.9%, and an overall accuracy of 95.1%, with a Youden index of 0.902 (Table 3). Cross-reactivity testing using sera from patients with other parasitic infections revealed no detectable cross-reactivity, confirming the high analytical specificity of the in-house ELISA.

**Table 3:** Diagnostic performance of the in-house 12-KD AgB ELISA.

Method/criterion	Cut-off	TP	FN	TN	FP	Sensitivity (%)	Specificity (%)	PPV	NPV	Accuracy	Youden index
Youden index (optimal)	0.256	46	5	50	0	0.902 (90.2%)	1.000 (100%)	1.000 (100%)	0.909 (90.9%)	0.951 (95.1%)	0.902
Maximum Accuracy	0.256	46	5	50	0	0.902 (90.2%)	1.000 (100%)	1.000 (100%)	0.909 (90.9%)	0.951 (95.1%)	0.902
Max (Sensitivity + Specificity)	0.256	46	5	50	0	0.902 (90.2%)	1.000 (100%)	1.000 (100%)	0.909 (90.9%)	0.951 (95.1%)	0.902
Commercial ELISA kit	0.31	47	4	50	0	0.922 (92.2%)	1.000 (100%)	1.000 (100%)	0.926 (92.6%)	0.960 (96.0%)	-

TP: True positive; FN: false negative; TN: true negative; FP: false positive; PPV: positive predictive value; NPV: Negative predictive value.

### Diagnostic performance with the commercial ELISA Kit

Parallel testing of all sera using the commercial ELISA kit revealed a mean OD_450_ of 0.995 ± 0.341 (range: 0.272–1.932) for CE-positive sera and 0.058 ± 0.010 (range: 0.044–0.074) for healthy controls, with the difference between groups being highly significant (*p* < 0.001). According to the kit instructions, the cut-off was calculated as the mean of negative controls plus 0.25, yielding 0.3102. For interpretation, OD values 10% above the cut-off (≥ 0.341) were considered positive, whereas OD values 10% below the cut-off (≤ 0.2792) were considered negative. Based on this threshold, 47 out of 51 CE patients were positive, while 4 patients with OD values below 0.341 were classified as negative. All 50 healthy controls were negative, resulting in a sensitivity of 92.2%, a specificity of 100%, a positive predictive value of 100%, a negative predictive value of 92.6%, and an overall accuracy of 96%.

### Detection of circulating cfDNA of *E. granulosus* s.s. by PCR

The extraction of cfDNA from plasma samples resulted in concentrations ranging from 5.2 to 42.8 ng/µL, with a mean of 21.6 ± 9.4 ng/µL and acceptable purity measured at A_260_/A_280_ = 1.78 ± 0.06.

PCR amplification using *E. granulosus*-specific COX1 primers generated a distinct ~ 450 bp band in 45 out of 51 (88.2%) confirmed CE cases (Supplementary 3). The remaining 6 samples (11.8%) showed no detectable amplification, indicating PCR-negative results. No amplification was observed in any healthy or disease-control samples, confirming the complete analytical specificity of the assay.

### Diagnostic performance and agreement analysis of in-house 12-kDa AgB ELISA, commercial ELISA kit, and PCR

The diagnostic performance and agreement between the in-house 12-kDa AgB ELISA, the commercial ELISA kit, and PCR were evaluated in 51 confirmed CE patients. OD values of the two ELISA methods were positively correlated (Spearman’s ρ = 0.872, *p* < 0.001), indicating a strong correlation between the optical densities. Binary classification (positive/negative) showed very good agreement between the in-house ELISA and the commercial kit (Cohen’s κ = 0.878), between the in-house ELISA and PCR (κ = 0.81), and between the commercial kit and PCR (κ = 0.78). These results demonstrate that both ELISA methods are highly consistent with each other and with PCR, confirming their reliability for the serological diagnosis of CE (Table 4).

**Table 4:** Comparison of diagnostic performance of cfDNA-PCR, in-house 12-kDa AgB ELISA, and commercial ELISA kit in confirmed CE cases.

Diagnostic Method	Sensitivity (%)	Specificity (%)	Accuracy (%)	Cohen’s κ	*p*-value (for agreement)
cfDNA-PCR	88.2	100	91.3	0.78 ^*^	< 0.001
In-house 12-kDa AgB ELISA	90.2	100	95.1	0.81 ^**^	< 0.001
Commercial ELISA kit	92.2	100	96	0.878 ^***^	< 0.001

^***^Cohen’s κ between in-house AgB ELISA and Commercial ELISA kit.

^**^Cohen’s κ between in-house AgB ELISA and PCR.

^*^Cohen’s κ between Commercial ELISA kit and PCR.

### Quality control and reproducibility of the in-house ELISA

Intra-assay precision was determined by testing three control sera (high positive, low positive, and negative) in 10 consecutive replicates. The results were highly consistent, with low CV% for all controls. The average OD value was 1.760 for the high-positive control, 0.595 for the low-positive control, and 0.047 for the negative control. The relative CV% readings were 5.8%, 4.5%, and 7.8%. All CV% values were significantly lower than the acceptable threshold of 10%, indicating exceptional intra-assay precision. For inter-assay precision, the reproducibility across different runs was evaluated by testing the same three controls in duplicate over ten independent assays performed on different days. The inter-assay CV% values were determined as follows: 9.7% for the high-positive control, 8.1% for the low-positive control, and 13.2% for the negative control. All values were within the accepTable 15% range, showing that the assay is reliable and generates consistent findings over time (Table 5).

**Table 5:** Intra-assay and inter-assay precision of the 12-kDa AgB ELISA.

Control serum	Mean OD (450 nm)	Intra-assay CV% (*n* = 10) (%)	Inter-assay CV% (*n* = 10) (%)
High positive	1.760	5.8	9.7
Low positive	0.595	4.5	8.1
Negative	0.047	7.8	13.2

Assay linearity demonstrated a concentration-dependent response. As the serum was diluted, the OD decreased consistently. The OD value dropped from 2.150 at the highest concentration (200 µg/mL) to 0.126 at the lowest concentration (0.8 µg/mL). This graded reduction in signal with decreasing antigen concentration confirms the assay’s proper functionality and sufficient sensitivity across the tested range. The correlation between the expected relative concentrations (log-transformed) and the observed OD values yielded a coefficient of determination (R^2^) of 0.993, indicating a highly linear relationship across the working range of the assay.

## Discussion

The present study assessed the diagnostic performance of an in-house ELISA based on the purified 12-kDa subunit of AgB from *E. granulosus* s.s. and compared its efficacy with a commercial IgG ELISA kit and a cfDNA-based PCR assay. To our knowledge, this is the first report that directly compares a genotype-matched AgB subunit ELISA with both a commercial serological kit and a molecular cfDNA assay for the diagnosis of human CE.

The findings demonstrate that the 12-kDa AgB-based ELISA exhibits high diagnostic efficiency, reinforcing the established view that AgB and its subunits remain among the most reliable serological markers for CE. The rationale for prior molecular genotyping of the local isolate was to ensure antigenic compatibility with the predominant circulating genotype (G1), thereby enhancing the clinical relevance and sensitivity of the in‑house test in the study region^24–26^. Recent studies have demonstrated that the application of native AgB derived from specific genotypes, such as *E. granulosus* s.s. (G1–G3), can influence test performance. For instance, Sharifi et al.^26^ reported that using AgB achieved up to 100% sensitivity in ELISA for detecting antibodies against G1–G3 genotypes. Similarly, Sadjjadi et al.^24^ confirmed the high diagnostic ability of AgB from *E. granulosus*. Therefore, the initial genotyping step in our protocol was not merely a taxonomic exercise but a critical strategy to align the diagnostic antigen with the local epidemiological profile, maximizing serological detection. This practice is particularly recommended in endemic regions hosting multiple genotypes to ensure broad reactivity and optimal sensitivity of serological assays^24–26^.

The present study demonstrated that the ELISA kit developed using purified AgB exhibits sensitivity and specificity comparable to, or exceeding, those reported in previous studies using the same antigen^24–27^. Evidence suggests that targeting the 12-kDa subunit specifically can improve diagnostic accuracy by minimizing cross-reactive reactions^17^. The high molecular purity of this subunit, confirmed by SDS-PAGE, likely contributes to the strong specificity observed. This aligns with recommendations from Bauomi et al., who emphasize that antigenic homogeneity and successful purification are central to improving ELISA performance^28^.

The comparison with a commercial ELISA kit further underscores the diagnostic value of the purified 12-kDa subunit. Although commercial assays offer the advantage of standardization, several reports, including those by Fotoohi et al.^29^ and Mohammadzadeh et al.^25^, suggest that in-house tests using refined AgB preparations often outperform commercial systems in sensitivity while maintaining acceptable specificity. The superior performance of our assay likely reflects both its purified antigenic composition and its genotype compatibility^25,29^.

Beyond native antigens, recombinant subunits of AgB have emerged as promising tools for serodiagnosis, offering advantages in standardization and large-scale production. Studies evaluating recombinant AgB subunits, such as the 8, 12, and 16 kDa subunits, have reported high diagnostic performance. For instance, Savardashtaki et al.^30^ found that a recombinant B8/2 subunit ELISA achieved a sensitivity of 90% and specificity of 93.5%, showing good agreement with native AgB and commercial kits. Similarly, Khatami et al.^31^ demonstrated that combining recombinant AgB8/1 and AgB8/2 subunits increased test sensitivity to 96.7%, highlighting the potential of multi-subunit recombinant formulations. Earlier work by Abdi et al.^32^ on a recombinant 12-kDa subunit showed excellent sensitivity (96%) and specificity (97%), performance comparable to that of cyst fluid. In another study, the same group reported that a recombinant 16 kDa subunit exhibited a sensitivity of 93.5% and a specificity of 95.6% for diagnosing human hydatidosis^33^. These findings collectively suggest that well-characterized recombinant subunits can match or approach the diagnostic efficacy of purified native antigens.

Although statistical analysis revealed no significant association between cyst stage and serological outcome, a clinically relevant pattern emerged. All seronegative results from both ELISAs were confined to patients with active or transitional cysts (CE1–CE3), whereas no seronegativity was observed in inactive cyst stages (CE4–CE5). The seronegative results in early active cysts could reflect a delayed or insufficient humoral immune response during the initial phases of infection, where antigen exposure may not yet have reached levels necessary to elicit a detectable antibody response. In contrast, the sustained seropositivity in inactive cysts suggests persistence of the antibody response, possibly driven by residual antigenic stimulation, prolonged IgG half-life, or antigen release during cyst involution^12,13,34^.

In addition to serology, the study evaluated cfDNA as a molecular diagnostic tool. Although cfDNA assays have gained attention in parasitic diseases due to their potential for early detection and treatment monitoring, their performance in CE has been inconsistent^18,35^. The cfDNA results obtained in this study align with findings from prior academic research, demonstrating moderate sensitivity that is influenced by factors such as sample type, cyst characteristics, and methodological variables^36–39^. Plasma generally yielded better detection rates than serum and urine, reflecting differences in cfDNA concentration, as noted by previous studies. Moreover, studies employing short amplicons, repetitive genomic targets, or nested PCR designs have typically achieved higher sensitivity, whereas the single-target PCR used in the present study was optimized for specificity rather than maximal detection. The overall performance of the cfDNA assay did not surpass that of ELISA, supporting the prevailing view that molecular detection of parasite-derived cfDNA is currently better suited as a complementary rather than primary diagnostic approach for CE^36,38,40^.

This study offers valuable insights into the diagnostic utility of the 12-kDa AgB subunit and its potential for enhancing serodiagnosis in endemic settings. By directly comparing three distinct diagnostic modalities an in-house AgB ELISA, commercial ELISA kits, and cfDNA PCR we provide a comprehensive evaluation rarely addressed simultaneously in prior research. AgB-encoding genes have been reported to show genetic polymorphism among *E. granulosus* genotypes, with suggested sequence differences between *E. granulosus* s.s. (G1–G3) and other genotypes such as G6/G7^16,41,42^. These differences may influence serodiagnostic performance, and some studies have indicated that genotype-matched antigens can improve detection sensitivity^26,43^. Thus, the use of a genotype-matched antigen could further strengthen the relevance of our findings for regions where *E. granulosus* s.s. predominates.

However, it is crucial to acknowledge the inherent limitations of serological tests in this context, which may impact the interpretation of our results. As noted in recent literature^44^, serological assays frequently exhibit suboptimal sensitivity and specificity, leading to low predictive values and potentially unreliable estimates of true disease prevalence. Various factors, including cyst stage and localization, cyst number and size, parasite genotypes, and endemic co-infections, can all influence serological responses, complicating definitive interpretation of seroprevalence data. Consequently, these limitations in our study may have contributed to possible false negatives or positives, particularly given the variability in individual immune responses. While serology remains a useful tool, its application, especially in endemic regions with diverse clinical presentations, warrants cautious interpretation. Future efforts should concentrate on developing standardized recombinant subunits to enhance the adoption, reproducibility, and accessibility of AgB-based tests for routine diagnostics in endemic regions. Additionally, exploring DNA extracted directly from patient cyst tissue may offer a more sensitive and stable template for molecular confirmation, potentially mitigating limitations inherent in current serological and plasma cfDNA-based methods.

## Conclusion

The in‑house ELISA developed in this study, based on the purified 12-kDa subunit of AgB from a genotype‑matched *E. granulosus* s.s. isolate, demonstrates excellent diagnostic performance, rivaling that of a commercial kit and a cfDNA‑PCR assay. Its high specificity, minimal cross‑reactivity, and sustained sensitivity even in inactive cysts highlight its utility as a robust and cost‑effective diagnostic tool. For clinically challenging cases, especially in early infection stages, a combined diagnostic approach utilizing initial ELISA screening followed by cfDNA testing and imaging is recommended to maximize diagnostic accuracy.

## Supplementary Information

Below is the link to the electronic supplementary material.


Supplementary Material 1



Supplementary Material 2


## Data Availability

All nucleotide sequences produced during this investigation have been submitted to the GenBank database. They are now publicly available with the following accession numbers: PX735995 for the cox1 gene and PX753202 for the nad1 gene. Links to the respective GenBank entries are provided: cox1 (https://www.ncbi.nlm.nih.gov/nuccore/PX735995) and nad1 (https://www.ncbi.nlm.nih.gov/nuccore/PX753202).

## Abbreviations

CE: Cystic echinococcosis
PCR: Polymerase Chain Reaction
ELISA: Enzyme-linked immunosorbent assay
AgB: Antigen B
cfDNA: Cell-free DNA
