# Supplementary material for: High efficacy of an in-house antigen B ELISA for diagnosing human cystic echinococcosis compared with a commercial kit and a cell-free DNA assay
Source: Sci Rep. 2026 Jun 16;16:27477. doi: 10.1038/s41598-026-58120-x (PMC13534585; doi:10.1038/s41598-026-58120-x)
Supplement: Supplementary file 1 [file 41598_2026_58120_MOESM2_ESM.docx]

**Supplementary 1.** PCR amplification of *E. granulosus* targets from protoscoleces. **(A)** Amplification of the ~450 bp COX1 fragment. Lane M: 100 bp molecular weight marker; N: negative control; P: positive control; S: protoscolex sample. **(B)** Amplification of the ~400 bp nad1 fragment. Lane order is identical to panel A.

**Supplementary 2.** SDS-PAGE analysis of purified AgB. The gel was run under reducing conditions on a 4–20% Tris‑Glycine gradient gel. Lane M: molecular weight marker (kDa values indicated on the left). Lanes 1–4: purified AgB samples showing a prominent band at approximately 12-kDa, corresponding to the principal subunit of AgB.

**Supplementary 3.** Agarose gel (1.5%) analysis of *E. granulosus* cox1 PCR amplicons. Electrophoresis confirms the amplification of the target gene from human plasma samples. Lane assignments: M, 100 bp molecular weight marker; N, negative control; P, positive control; Lanes S1-S9 represent positive patient samples.
